# 20 000 - 3 000 Traffic fatalities per year - Milestones of Road Safety in Germany

**Published:** 2019-08

**Authors:** Guenter Lob

**Affiliations:** ^*a*^Department of Trauma Surgery, Ludwig-Maximillians-University Munich, Germany.

## Abstract

Up to 1970 the number of traffic fatalities was rising from year to year. The burden for families as well as for the society was tremendous. Therefore, a strong movement was started within our society, to stop this deadly war on our streets. The government published laws: Speed limit in town and outside, seatbelts obligatory for all passengers, airbags in all cars, alcohol restriction, and mandatory helmets for motorcyclist and etc. Law enforcement was implemented and executed by trained traffic police.

The car industry detected, that safety is a marketing tool to sell cars in Germany as well as in other countries. Research programs were started to develop the most effective safety standard, which was then passed into law in Germany and the European Union.

The ADAC a NGO (German Automobile Club) started a SAR Helicopter Service, today flying overall in Germany. The SAR helicopter are linked to the EMS on ground. For an effective medical treatment the trauma hospitals work together in a Trauma Network, which is organized and certified by the German Trauma Society a NGO (DGU). The data for the Trauma Network are generated by the Trauma Register of the DGU, which collects specific data since 1993. Children in the schools were trained by specialized police officers to travel safely on all streets. Television shows safety warning and injury prevention clips at the best sending time.

Most accidents occur due to human errors. To canalize the human factor, a mosaic of activities must be implemented over a long time. The people must understand and well accept the necessary safety procedures. With the help of all citizen we are able to reduce the burden of traffic accidents and with a sophisticated technology, zero traffic fatalities might be possible in the future.

**Keywords:**

Road Safety, Human Errors, Injury

